# Optimization of a Totally Fiber-Reinforced Plastic Composite Sandwich Construction of Helicopter Floor for Weight Saving, Fuel Saving and Higher Safety

**DOI:** 10.3390/polym13162735

**Published:** 2021-08-15

**Authors:** Alaa Al-Fatlawi, Károly Jármai, György Kovács

**Affiliations:** 1 Faculty of Mechanical Engineering and Informatics, University of Miskolc, Egyetemváros, 3515 Miskolc, Hungary; vegyalaa@uni-miskolc.hu (A.A.-F.); jarmai@uni-miskolc.hu (K.J.); 2 Faculty of Mechanical Engineering, University of Kufa, Al-Najaf 54001, Iraq

**Keywords:** composite sandwich structure, FRP honeycomb core, FRP face sheets, helicopter floor, material constituents, structural optimization, Interior Point Algorithm, GRG Nonlinear Algorithm

## Abstract

The application of fiber-reinforced plastic (FRP) composites as structural elements of air vehicles provides weight saving, which results in a reduction in fuel consumption, fuel cost, and air pollution, and a higher speed. The goal of this research was to elaborate a new optimization method for a totally FRP composite construction for helicopter floors. During the optimization, 46 different layer combinations of 4 different FRP layers (woven glass fibers with phenolic resin; woven glass fibers with epoxy resin; woven carbon fibers with epoxy resin; hybrid composite) and FRP honeycomb core structural elements were investigated. The face sheets were composed of a different number of layers with cross-ply, angle-ply, and multidirectional fiber orientations. During the optimization, nine design constraints were considered: deflection; face sheet stress (bending load, end loading); stiffness; buckling; core shear stress; skin wrinkling; intracell buckling; and shear crimping. The single-objective weight optimization was solved by applying the Interior Point Algorithm of the Matlab software, the Generalized Reduced Gradient (GRG) Nonlinear Algorithm of the Excel Solver software, and the Laminator software. The Digimat-HC software solved the numerical models for the optimum sandwich plates of helicopter floors. The main contribution is developing a new method for optimizing a totally FRP composite sandwich structure—due to its material constituents and construction—that is more advantageous than traditional helicopter floors. A case study validated this fact.

## 1. Introduction

At present, applications of new advanced materials and constructions, as well as innovative and environmentally friendly technologies, are needed both in the manufacturing and transport sectors to increase companies’ competitiveness and provide sustainability [1,2,3]. The most expensive and environmentally damaging transport mode is air transport. Therefore, the main aims are reducing fuel consumption and reducing fuel costs, in addition to achieving less environmental damage and improvements in the efficient and safe operation of air transport [4,5,6].

The importance of this research topic is that—among air vehicles—helicopters, in particular, have a key role in several special, complex, and risky situations. Only helicopters can perform many important and dangerous tasks, i.e., air ambulance, fire fighting, aerial surveillance, and rescue tasks. Therefore, there are a lot of requirements, especially for helicopters, which are as follows: low weight; high speed; easy and safe maneuverability; cost-efficient operation (low fuel consumption); and safe transportation (e.g., reliability, crashworthiness) [7,8]. Crashworthiness is an important requirement relating to helicopters because the structure of helicopters has to withstand an impact and protect the helicopters’ occupants. Thus, the floor structure of helicopters should be designed to withstand loads and accelerations on the human body during a crash landing. Sometimes helicopters crash due to human errors or technical failures. The design of helicopters has always remained a top priority to avoid structural failures. Crashes of helicopters can be reduced by using energy-absorbing materials or structures [9,10]. Several articles are available on the topic of crashworthy design and energy absorption mechanisms for helicopter structures [11,12,13,14].

The application of advanced composite materials can fulfill the requirements mentioned before relating to helicopters during the design and manufacturing of helicopters’ structural elements, e.g., the floor of helicopters. The reason for this is that composites have more advantageous characteristics than conventional materials [15,16]. Composite materials have a low density, resulting in a reduction in weight, which causes, on the one hand, reduced fuel consumption, fuel cost reduction, and less air pollution; on the other hand, it can allow for higher speeds of helicopters. Furthermore, composite structures have a high strength, good vibration damping, corrosion and chemical resistance, fire resistance, and good thermal insulation [17,18]. The above-mentioned properties of composite materials provide a wide range of applications of these advanced materials, primarily as structural elements of air vehicles, e.g., helicopters.

The most often used types of composite materials are fiber-reinforced plastic (FRP) composites. FRP composites consist of two components: (1) a matrix (generally resins) and (2) a strengthening component (generally fibers). Fibers provide the strength of composite materials. The matrix holds the fibers and protects them from negative environmental effects. There are many types of available fibers and matrix phases. The combinations of these different fibers and matrices are various and provide the tailorability of the materials for a given engineering application [19].

In practice, the most commonly used FRP composites combine the following fibers: carbon, glass, aramid, etc., and the following matrices: epoxy, phenol, etc. Epoxy resin is a polymer with good mechanical properties, excellent environmental resistance, and simple processing. Phenolic resin is a synthetic polymer with good chemical, fire, and thermal resistances, low toxic emissions, and easy processing [20,21]. Many types of synthetic fibers are utilized to reinforce plastic materials such as glass fiber and carbon fiber. Glass fiber is the most widely utilized for reinforcement and has the lowest cost. Carbon fiber has high strength-to-weight ratios and is utilized in many applications, especially aerospace applications, despite its high cost [22,23].

1. FRP sandwich structures are some of the most widely applied structural elements in engineering applications. FRP sandwich structures are built from FRP face sheets (e.g., laminated plates) and core materials (e.g., honeycomb, foam). These structures provide a high strength and stiffness, easy assembly, and excellent tailorability [24,25,26].
2. Many relevant publications are available on the design and optimization procedures of composite sandwich structures to construct optimal structures that provide a high stiffness and strength, in addition to a low weight and cost [27,28,29]. Heimbs et al. found that the mechanical behavior of the sandwich construction consists of a folded core made of carbon fiber-reinforced plastic; furthermore, they discussed the development of the folded core models in the LS-DYNA FE software. The validation of models was performed by optimizing the LS-OPT software concerning core manufacturer experimental data [30,31]. Bisagni et al. elaborated an optimization method under crashworthiness conditions for a typical helicopter subfloor made of aluminum alloy [32]. Adel and Steven minimized the single-objective function and multi-objective functions for foam sandwich plates with hybrid composite face sheets subjected to bending and torsional stiffness constraints [33].
3. Some articles discussed experimental and computational analysis to assess foam-formed materials’ sound insulation capabilities and applied the gray relational analysis method and multi-objective particle swarm optimization algorithm to develop the acoustic performances of foam composites [34,35,36]. Khan et al. described the improvement models of the smallest cell for quantifying the deformation and failure modes for a core structure under static loadings [37].
4. Different techniques and methods have been introduced in the literature to solve optimization problems in various composite structures [38,39,40,41]. Furthermore, many software applications (e.g., Matlab, Abaqus) have become common for structural optimization. The finite element software applications are often used to numerically solve differential equations during structural analysis [42,43,44]. Khalkhali et al. used a modified genetic algorithm to solve the weight and the deflection functions of sandwich panels with a corrugated core [45]. Corvino et al. introduced a procedure for multi-objective optimization based on genetic algorithms with the ANSYS software [46].
5. Based on the synthesis of the existing literature, it can be concluded that although there are several design and optimization methods available for optimization of sandwich structures, no method can be found relating to a totally FRP sandwich (both the face sheets and the honeycomb core are FRP materials) construction. Therefore, the newly elaborated optimization method fills a gap in this research field.

This research aimed to elaborate the optimization method for a totally FRP composite—FRP face sheets with an FRP honeycomb core—sandwich structure for the floor of helicopters. The goal of the optimization was the determination of the optimal material constituents and structure of the helicopter floor that provide the minimal weight. During the optimization, 46 different layer combinations of 4 different types of FRP layers (1. woven glass fibers with phenolic resin; 2. woven glass fibers with epoxy resin; 3. woven carbon fibers with epoxy resin; and 4. hybrid composite layers) and FRP honeycomb core structural elements were investigated. The face sheets were composed of a different number of layers with cross-ply, angle-ply, and multidirectional fiber orientations.

The authors elaborated a single-objective weight optimization method by applying nine design constraints, which are the following: deflection; face sheet stress (bending load and end loading); stiffness; core shear stress; buckling; skin wrinkling; shear crimping; and intracell buckling. The optimization was solved by applying the Interior Point Algorithm of the Matlab software, the Generalized Reduced Gradient Nonlinear Algorithm of the Excel Solver software, and the Laminator software. The numerical models for the optimal sandwich structures of helicopter floors were constructed by applying the Digimat-HC software. In addition, the safety factors were calculated for all of the nine design constraints used during the optimization of the helicopter floor.

The main contribution is developing a new method for optimizing a totally FRP composite sandwich structure—due to its material constituents and construction—that is more advantageous than traditional helicopter floors. Furthermore, in the newly elaborated optimization method, nine design constraints are considered, while the optimization methods available in the existing literature generally apply only three–four constraints. The larger number of design constraints provides higher safety of the optimal sandwich structure, and thereby a safer helicopter operation. It can be concluded that the newly designed totally FRP construction—due to its low density—provides a higher weight saving, and thereby lower fuel consumption, a lower fuel cost, and less environmental damage, than conventional structures. Consequently, the optimal totally FRP structures—designed by our new method—can be widely used in practice in different engineering applications, e.g., structural elements of transport vehicles (ship decks, components of road vehicles, etc.). A case study validated the efficiency and practical applicability of our newly elaborated method.

## 2. Materials and Methods—Structure and Material Constituents of the Newly Designed Helicopter Floor

**Figure 1:**
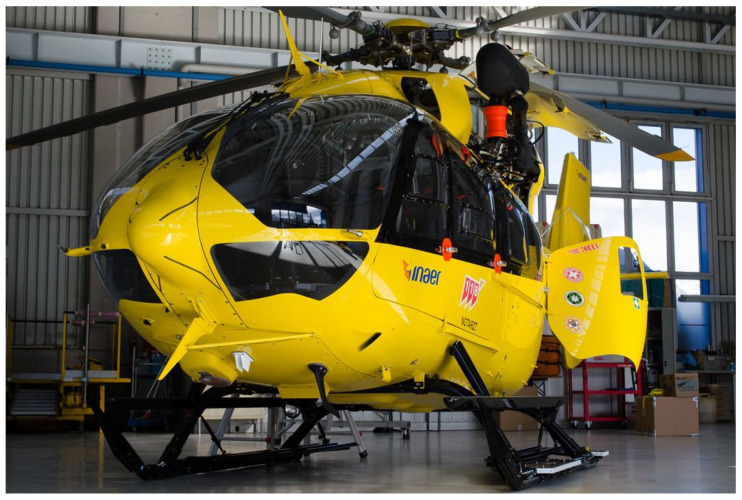
Airbus helicopter [47].

The newly designed lightweight sandwich plate of the helicopter floor consists of an FRP honeycomb core and various types of face sheets including: (1) woven glass fiber with phenolic resin, (2) woven glass fiber with epoxy resin, (3) woven carbon fiber with epoxy resin, and (4) hybrid composite layers (combined layers of woven glass fiber epoxy with resin, and woven carbon fiber with epoxy resin), with sets of different fiber orientations: (1) cross-ply, (2) angle-ply, and (3) multidirectional. The Airbus helicopter floor structure, shown in Figure 1, can be developed using sandwich technology [47].

The floor panel of a helicopter has dimensions of 1500 by 825 mm and is self-supporting, i.e., there are no external support frames except around the edges of the floor. The floor plate is subjected to a uniform distributed pressure of p = 1500 kg/m^2^ times 4.5 g acceleration and deforms by $\delta_{max}$ = 10 mm (see Table 1). There are simply supported boundary conditions for the plate of the helicopter floor, and l/b = 1.8 (see Table 2).

### 2.1. Structure of the Newly Designed Helicopter Floor Panel

**Figure 2:**
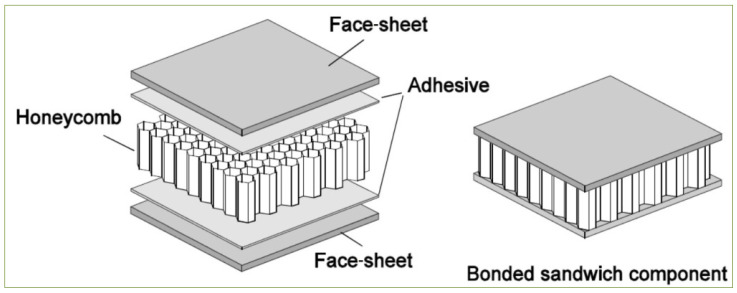
Construction of honeycomb core sandwich plate.

FRP sandwich plates were designed to be lightweight and have a relatively high stiffness-to-weight ratio. The FRP composite sandwich plates consisted of two FRP outer face sheets (upper and lower) separated by a thicker FRP honeycomb core and bonded together by an adhesive. The result of the high stiffness comes from the distance between the face sheets, which bear the force, and the light weight of the sandwich plate is due to the ligh weight of the honeycomb core. The design properties for the composite honeycomb core make it perfect for many industrial applications such as helicopter floors (see Figure 2).

#### 2.1.1. Face Sheets of the Sandwich Plate

**Figure 3:**
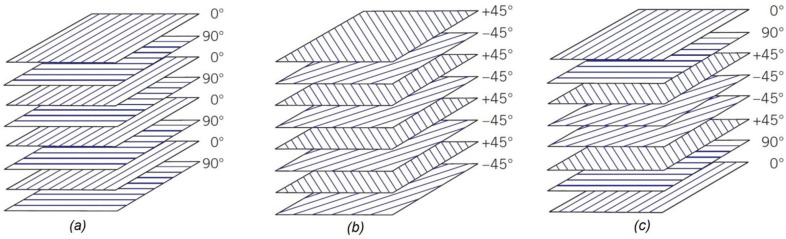
Composite laminated lay-ups. (**a**) Cross-ply, (**b**) angle-ply, and (**c**) multidirectional (0°, 90°, and ±45°).

Figure 3 shows three classes of composite laminated plates used in this paper, which are cross-ply, angle-ply, and multidirectional. The mechanical properties of the facing materials are shown in Table 3. The layers of the face sheets are the products of the Hexcel Composites Company.

#### 2.1.2. Honeycomb Core of the Sandwich Plate

**Figure 4:**
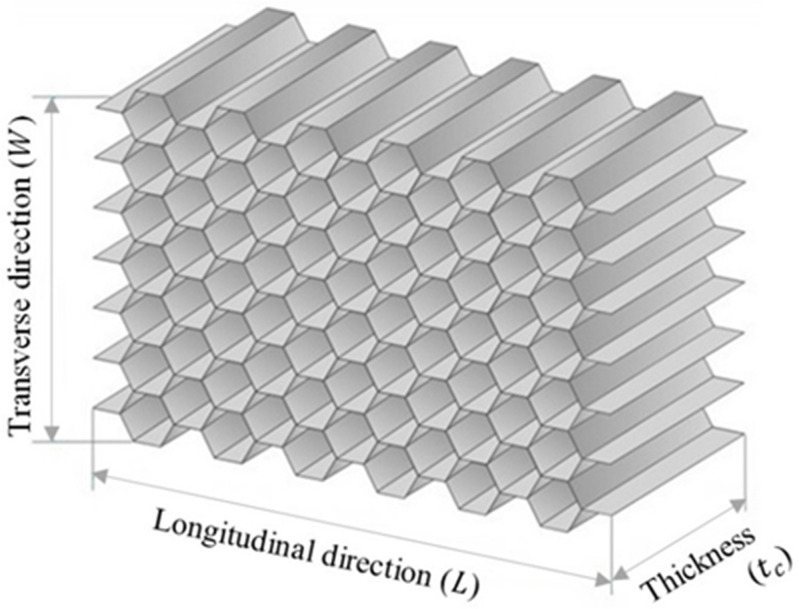
Honeycomb cell configurations (hexagonal core).

The standard hexagonal honeycomb core is the primary and most popular cellular honeycomb shape and is currently available in metallic and composite materials (see Figure 4).

The mechanical properties of the FRP honeycomb core satisfy the requirements of most airframe manufacturers’ specifications, as shown in Table 4. The honeycomb core is the product of the Hexcel Composites Company.

## 3. Single-Objective Optimization Methods

### 3.1. Weight Objective Function

The total weight of the sandwich structure is

(1) $$W_{t}=W_{f}+W_{c}=2\;\rho_{f}lbt_{f}+\rho_{c}lbt_{c}$$

where $t_{f}=N_{l}t_{l}$; indexes: *f*—face; *c*—core.

The weight equation for the hybrid composite face sheets is

(2) $$W_{t}=W_{f}+W_{c}=2(W_{f,g}+W_{f,cr})+W_{c}=2(\rho_{g}N_{g}t_{g}+\rho_{cr}N_{cr}t_{cr})lb+\rho_{c}lbt_{c}$$

### 3.2. Design Variables

The composite honeycomb core thickness $t_{c}$ and face sheet thickness $t_{f}$ for the sandwich plate of the helicopter floor have to be limited:

(3) $$1\;\mathrm{mm}\leq t_{c}\leq 100\;\mathrm{mm}$$

(4) $$0.5\;\mathrm{mm}\leq t_{f}\leq 2\;\mathrm{mm}$$

where $t_{f}=N_{l}t_{fl}$;

$N_{l}$—number of layers in the laminate;

$t_{fl}$—thickness of one layer.

### 3.3. Design Constraints

#### 3.3.1. Stiffness

The bending stiffness constraint for the sandwich plate of the helicopter floor with composite material face sheets is

(5) $$D_{11,x}=D_{11}/\left(1-\nu_{12}^{f}\;\nu_{21}^{f}\right)\geq D_{min}=\frac{K_{b}pl^{4}}{\delta }$$

where $D_{11}=0.5d^{2}A_{11}^{f}+2D_{11}^{f}+2dB_{11}^{f}$, $\nu_{12}^{f}=A_{12}^{f}/A_{22}^{f}$, $\nu_{21}^{f}=A_{12}^{f}/A_{11}^{f}$, and $d=t_{f}+t_{c}$.

The shear stiffness for the sandwich plate of the helicopter floor with composite material face sheets is

(6) $${\tilde{S}}_{11}=\frac{d^{2}}{t_{c}}\frac{E_{c}}{2\;\left(1+\nu_{c}\right)}$$

The sandwich plate of the helicopter floor’s calculated stiffness should be greater than the minimum stiffness, computed using the data presented in Table 1 and Table 2.

#### 3.3.2. Deflection

The deflection constraint for the sandwich plate of the helicopter floor is

(7) $$\delta_{max}\geq \delta =\frac{K_{b}pl^{4}}{D_{11,x}}+\frac{K_{s}pl^{2}}{{\tilde{S}}_{11}}$$

The maximum deflection of the sandwich plate of the helicopter floor $\delta_{max}$ that is provided in Table 1 should be higher than the calculated deflection δ.

#### 3.3.3. Skin Stress

The skin stress constraint for the sandwich plate of the helicopter floor is

(8) $$\sigma_{f,x}\geq \sigma_{f}=\frac{M}{dt_{f}b}$$

$\sigma_{f,x}$—yield strength of the FRP face sheets in the x direction (calculated by the Laminator software);

$\sigma_{f}$—calculated skin stress.

#### 3.3.4. Core Shear Stress

The core shear stress constraint can be calculated as

(9) $$\tau_{c,y}\geq \tau_{c}=\frac{F}{db}$$

$\tau_{c,y}$—shear stress of the composite honeycomb core in the transverse direction (Table 4);

$\tau_{c}$—calculated core shear stress.

#### 3.3.5. Facing Stress (End Loading)

The facing stress constraint can be calculated as

(10) $$\sigma_{f,y}\geq \sigma_{f}=\frac{P}{2t_{f}b}$$

$\sigma_{f,y}$—yield strength of the composite face sheets in the y direction (calculated by the Laminator software);

$\sigma_{f}$—calculated facing stress.

#### 3.3.6. Buckling

The buckling constraint for the sandwich plate of the helicopter floor with composite material face sheets is

(11) $$P_{b,cr}=\frac{\pi^{2}D_{11,x}}{\beta l^{2}+\frac{\pi^{2}D_{11,x}}{{\tilde{S}}_{11}}}\geq \frac{P}{b}$$

$P_{b,cr}$—computed load at which critical buckling occurs;

P/b—load per unit width.

#### 3.3.7. Shear Crimping

The shear crimping constraint can be calculated as

(12) $$P_{cr}=t_{c}G_{c}b\geq P$$

where $G_{c}=G_{w}$;

$P_{cr}$—computed load at which shear crimping occurs;

P—load utilized.

#### 3.3.8. Skin Wrinkling

The following skin wrinkling constraints can be calculated:

(13) $$\sigma_{wr,cr}=0.5\;\sqrt[{3}]{E_{f,x}\;E_{c}\;G_{c}}\;\geq \sigma_{f,x}$$

where $G_{c}=G_{L}$.

(14) $$\sigma_{wr,cr}=0.5\;\sqrt[{3}]{E_{f,y\;}E_{c}\;G_{c}}\;\geq \sigma_{f,y}$$

where $G_{c}=G_{W}$.

(15) $$P_{wr,cr}=2\sqrt{D_{11}^{f}\frac{E_{c}}{\left(t_{c}/2\right)}}\geq \frac{P}{b}$$

where $E_{f,x}=A_{11}^{f}\left(1-\nu_{12}^{f}\nu_{21}^{f}\right)/t_{f}$, $E_{f,y}=A_{22}^{f}\left(1-\nu_{12}^{f}\nu_{21}^{f}\right)/t_{f}$, and $E_{f}=\sqrt{E_{f,x}E_{f,y}}$.

The stress at which skin wrinkling $\sigma_{wr,cr}$ occurs is higher than the typical yield strength of the skin in the x direction $\sigma_{f,x}$ and in the y direction $\sigma_{f,y}$. It is calculated using the Laminator program.

$P_{wr,cr}$—load at which skin wrinkling occurs;

P/b—load per unit width.

#### 3.3.9. Intracell Buckling (Face Sheet Dimpling)

The intracell buckling constraint can be calculated as

(16) $$\sigma_{fib,cr}=\frac{2E_{f}}{\left(1-\nu_{12}^{f}\nu_{21}^{f}\right)}{\left[\frac{t_{f}}{s}\right]}^{2}\geq \sigma_{f,y}$$

where $E_{f}=\sqrt{E_{f,x}E_{f,y}}$;

$\sigma_{fib,cr}$—stress at which intracell buckling would happen;

$\sigma_{f,y}$—yield strength of the skin material (calculated by the Laminator software).

**Figure 5:**
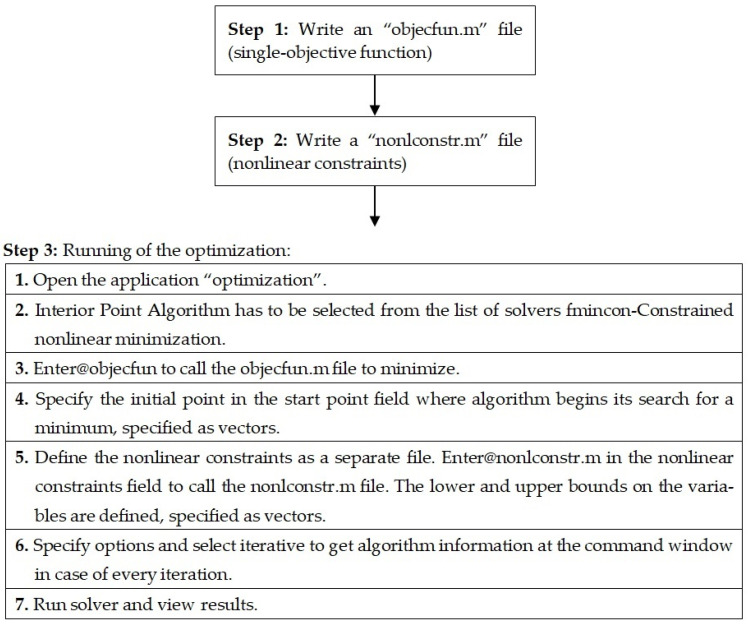
Flowchart for optimization procedure of single-objective function.

The Laminator program can solve the classical analysis of composite laminates. The procedure followed in the optimization to minimize the single-objective function is shown in Figure 5.

## 4. Results—Case Study for the Optimization of Helicopter Floor

The optimization results for the single-objective function include: $W_{min}$—minimum weight; $t_{c,opt}$—optimum core thickness; $t_{f,opt}$—optimum thickness of face sheets. The optimization problem is solved by applying both the Matlab software and the Excel Solver software.

The single-objective function was considered to decrease the weight objective function of the sandwich plate of the helicopter floor obtained utilizing the Excel Solver program (GRG Nonlinear Algorithm) and Matlab program (Interior Point Algorithm) for FRP face sheets and the FRP honeycomb core (hexagonal shape).

### 4.1. Weight Objective Optimization by Applying the Excel Solver Software for Sandwich Structure of the Helicopter Floor

Table 5 shows the optimal results of the weight objective function for the sandwich plate of the helicopter floor consisting of a composite honeycomb core with composite material face sheets obtained utilizing the Excel Solver program (GRG Nonlinear Algorithm).

### 4.2. Weight Objective Optimization by Applying the Matlab Software for Sandwich Structure of the Helicopter Floor

Table 6 shows the optimal results of the weight objective function for the sandwich plate of the helicopter floor consisting of a composite honeycomb core with composite material face sheets obtained by applying the Matlab software (Interior Point Algorithm).

### 4.3. Evaluation of the Optimization Results Achieved by Applying the Matlab and Excel Solver Software

Table 5 and Table 6 show the theoretical results for the optimum sandwich plate of a helicopter floor. The optimal results of the Matlab and Excel Solver programs relating to the thickness of the structural elements, as design variables $t_{f,opt}$ and $t_{c,opt}$ (Section 3.2), are the same. According to the data of Table 5 and Table 6, the optimum sandwich plate of a helicopter floor consisting of woven carbon fiber epoxy resin face sheets (two pieces of cross-ply layers) and an FRP honeycomb core ensures the minimum weight. The optimum thicknesses for the face sheets ($t_{f,opt}=0.6\;\mathrm{mm}$) and the optimum thickness of the core ($t_{c,opt}=95\;\mathrm{mm}$) are the same in the case of the optimization results achieved both by applying the Matlab and the Excel Solver software. Thus, the minimal weight of the optimal sandwich plate of a helicopter floor is 14.5 kg. Consequently, the optimal parameters ($t_{f,opt},t_{c,opt}$) and the calculated weight ($W_{min}$) of the newly developed optimal construction are the same in the case of the application of both the Matlab and the Excel Solver software (Table 5 and Table 6).

**Figure 6:**
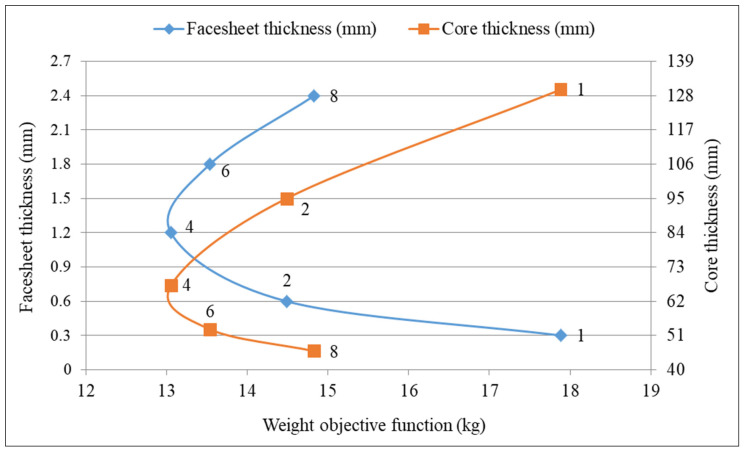
The relationship of the optimal thickness of face sheets and optimal thickness of the core in the case of the minimum weight.

Figure 6 graphically shows the relationship of the optimum thickness of the face sheets and the optimum thickness of the core in the case of the minimum weight based on the data of Table 5 and Table 6.

It can be concluded that the reliability of the newly elaborated optimization method (Section 3) is verified since the obtained optimal parameters of the new optimal construction are the same in the case of the application of both the Matlab and the Excel Solver software (Table 5 and Table 6).

Consequently, the developed optimal sandwich construction fulfills all of the nine design constraints (Section 3.3).

- The actual caltulated values for the optimal construction have to be less than the relevant maximum allowable values in the case of the following four design constraints to fulfill the requirements.
  1. Deflection (δ)—maximum allowable value: 25 mm/calculated value: 24.949 mm;
  2. Skin stress ($\sigma_{f,x}$)—maximum allowable value: 785.5 MPa/calculated value: 211.7 MPa;
  3. Core shear stress ($\tau_{c}$)—maximum allowable value: 2.28 MPa/calculated value: 0.338 MPa;
  4. Facing stress ($\sigma_{f,y}$)—maximum allowable value: 687 MPa/calculated value: 54 MPa.
- The actual caltulated values for the optimal construction have to be higher than the relevant minimum allowable values in the case of the following five design constraints to fulfill the requirements.
  5. Stiffness ($D_{11,x}$)—minimum allowable value: 174.6 kN·m/calculated value: 179.4 kN·m;
  6. Buckling ($P_{b}$)—minimum allowable value: 64.86 kN/m/calculated value: 766.61 kN/m;
  7. Shear crimping ($P_{cr}$)—minimum allowable value: 53.51 kN/calculated value: 7064.12 kN;
  8. Skin wrinkling ($P_{wr}$)—minimum allowable value: 64.86 kN/m/calculated value: 285.72 kN/m;
  9. Intracell buckling ($\sigma_{fib}$)—minimum allowable value: 785.2 MPa/calculated value 1296.9 MPa.

Based on the above-mentioned data, it can be summarized that the developed optimal sandwich construction fulfills all of the nine design constraints.

## 5. Further Advantages of the Newly Developed Totally Composite Sandwich Structure of the Helicopter Floor

### 5.1. Safety Factors Relating to the Design Constraints

The safety factor is very significant for design engineers and the most important quality to be considered when designing parts or structures. A fundamental equation to determine the safety factor is to divide the maximum stress or load by the typical stress or load. The safety factors for the optimum design constraints of the helicopter floor, which consists of a composite honeycomb core (fiberglass/phenolic resin) and the previously mentioned four different types of composite face sheets, are shown in Table 7.

### 5.2. Annual Fuel and Carbon Savings

According to the IATA (International Air Transport Association), the fuel weight needed to carry 1 kg of added weight per year is 200 kg, and the current cost per 1000 kg is about USD 993 from the Jet Fuel Price Monitor. Therefore, the cost to transport 1 kg of added weight for 1 year is about USD 199. The carbon generated per kilogram of fuel is about 3.1 kg, and the carbon generated to transport 1 kg/year is about 620 kg. The cost of CO_2_/ton is about USD 40, as shown in Table 8.

## 6. Numerical Analysis for Optimum Sandwich Plate of Helicopter Floor Using the Digimat-HC Program

**Figure 7:**
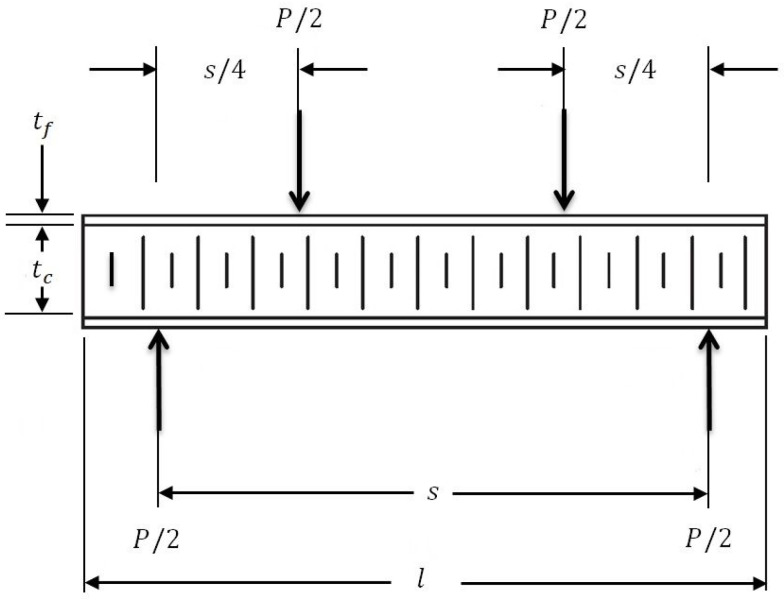
Four-point bending test setup of the honeycomb sandwich plate by the Digimat-HC program.

The Digimat-HC program is a multi-scale tool for modeling the four-point flexural test. The application of the software is precise and flexible for analysis of plates with honeycomb core structures. This study aimed to conduct a comparison of the numerical simulation between models of sandwich plates of the helicopter floor. The dimensions of the honeycomb sandwich models of the helicopter floor are shown in Table 9 (see Figure 7).

**Figure A1:**
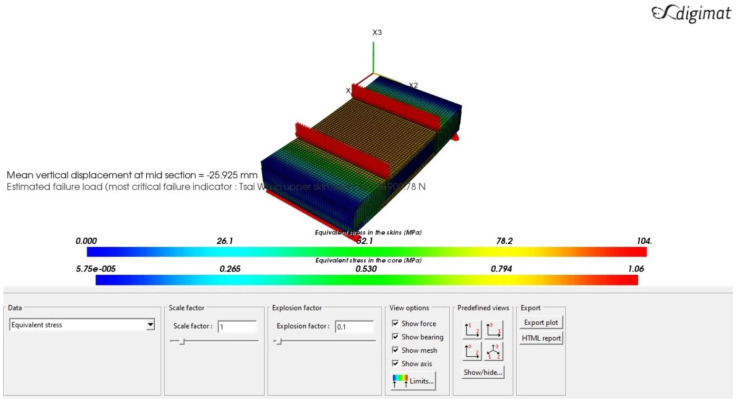
The four-point bending test for the sandwich plate of the helicopter floor numerical model. FRP honeycomb core ($t_{c}$ = 136 mm) and woven glass fiber with phenolic resin face sheets ($t_{f}$ = 1 mm).

**Figure A2:**
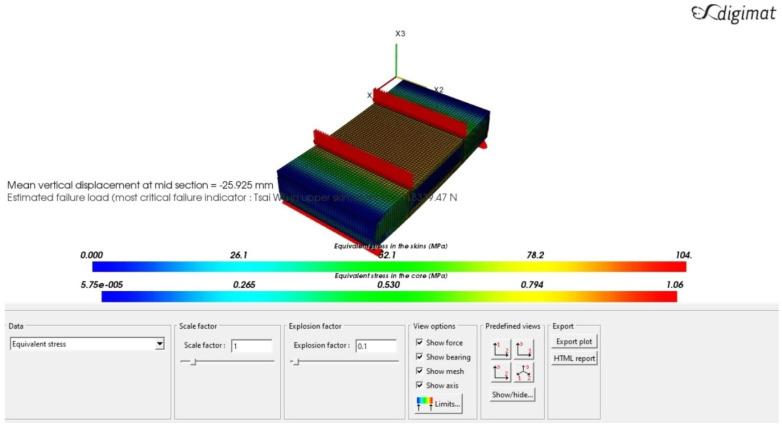
The four-point bending test for the sandwich plate of the helicopter floor numerical model. FRP honeycomb core ($t_{c}$ = 136 mm) and woven glass fiber with epoxy resin face sheets ($t_{f}$ = 1 mm).

**Figure A3:**
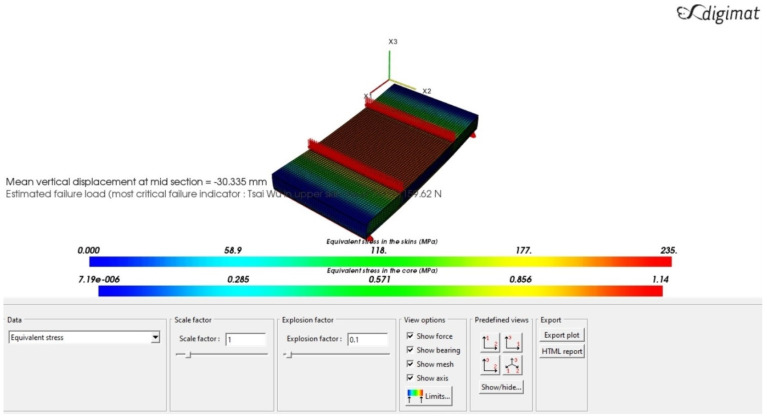
The four-point bending test for the sandwich plate of the helicopter floor numerical model. FRP honeycomb core ($t_{c}$ = 95 mm) and woven carbon fiber with epoxy resin face sheets ($t_{f}$ = 0.6 mm).

**Figure A4:**
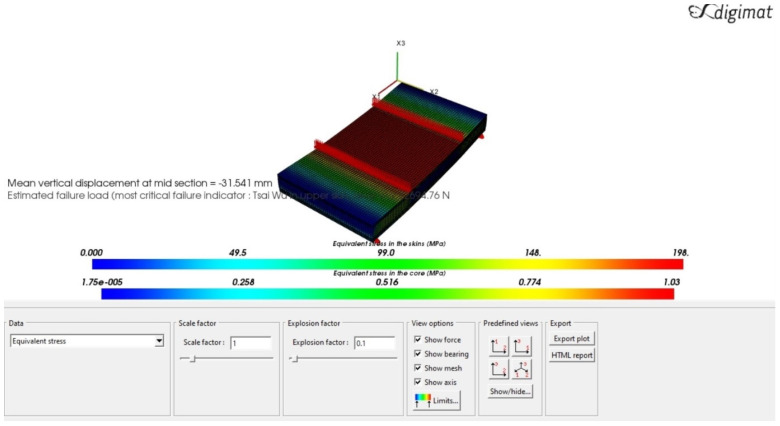
The four-point bending test for the sandwich plate of the helicopter floor numerical model. FRP composite honeycomb core ($t_{c}$ = 85 mm) and hybrid face sheets ($t_{f}$ = 1.1 mm).

- The four-point bending test was performed by applying the Digimat-HC software. The results of the simulation are the evaluation of the following parameters for the optimum sandwich plates of the helicopter floor and are shown in Table 10 (see Figure A1, Figure A2, Figure A3 and Figure A4 in Appendix B):
  - δ: vertical displacement of the structure at the mid-section;
  - $\sigma_{skin}$: equivalent skin stress;
  - $\tau_{c}$: equivalent core shear stress.

Figure A1, Figure A2, Figure A3 and Figure A4 in Appendix B show the graphical evaluation of the simulation four-point bending test relating to the honeycomb sandwich structures of the helicopter floor using the Digimat-HC software. The results of the simulation are the evaluation of the following parameters: (1) δ: vertical displacement of the structure at the mid-section; (2) $\sigma_{skin}$: equivalent skin stress; (3) $\tau_{c}$: equivalent core shear stress, for the optimum sandwich plates of the helicopter floor.

Table 10 shows the numerical results of the honeycomb sandwich structures of the helicopter floor using the Digimat-HC software.

## 7. Conclusions and Future Research

A new optimization method was elaborated for a totally FRP composite—both the face sheets and the honeycomb core are FRP composite materials—sandwich structure for the floor of helicopters. The optimal material constituents and structure of the helicopter floor can be determined by applying the new optimization method, which provides the minimal weight. In this method, nine design constraints were considered: deflection; face sheet stress (bending load and end loading); stiffness; buckling; core shear stress; skin wrinkling; intracell buckling; and shear crimping. During the optimization, the optimal material constituents of the FRP face sheets were defined from four different types of FRP layers (woven carbon fibers with epoxy resin; woven glass fibers with phenolic resin; woven glass fibers with epoxy resin; hybrid composite layers).

The practical applicability of the new optimization method was also validated by a case study. In the case study, the optimal totally composite sandwich plate for the helicopter floor is the construction of two layers of epoxy woven carbon fiber face sheets (fiber orientation is cross-ply (0°, 90°), face sheet thickness is 0.6 mm) and the FRP honeycomb core (95 mm thickness). The minimum weight of the optimal structure is 14.473 kg/piece.

The single-objective weight optimization was solved by applying the Interior Point Algorithm of the Matlab software and the Generalized Reduced Gradient Nonlinear Algorithm of the Excel Solver software. During the optimization of the face sheets, the Laminator software was also used. The numerical models for the optimum sandwich plates of the helicopter floor were constructed by the Digimat-HC simulation software.

The reliability and the applicability of the newly elaborated optimization method considering nine design constraints (Section 3) were verified since the obtained optimal results of the new optimal construction were the same in the case of the application of both the Matlab and the Excel Solver software (Table 5 and Table 6). Consequently, the developed optimal sandwich construction fulfills all of the nine design constraints (Section 4.3). Furthermore, the reliability of the elaborated optimization method was also verified by the application of the Digimat-HC finite element software (Section 6). The simulation results of the FE analysis of the optimal totally FRP construction confirm that the applied design constraints were fulfilled.

The main contribution of this research is developing a new method for optimizing a totally FRP composite sandwich structure—due to its optimal material constituents and construction—that is more advantageous than traditional helicopter floors. This means that the optimal newly designed totally FRP sandwich helicopter floor—due to its low density—provides a higher weight saving, and thereby lower fuel consumption, a lower fuel cost, and lower air pollution. Consequently, the optimal totally FRP structures—designed by our newly elaborated method—can be widely used in practice, i.e., as structural elements of vehicles.

Furthermore, it can be concluded that although there are several design and optimization methods available for the optimization of structural elements of air vehicles in the existing literature, no method can be found relating to a totally FRP sandwich construction. Therefore, the newly elaborated optimization method fills a gap in this research field.

It can be summarized that the determination of the appropriate material constituents and, at the same time, the construction of an adequate structure for a given engineering application are essential. In future research, the newly elaborated optimization method for totally FRP sandwich structures can be applied in further practical applications, e.g., different structural elements of road, water, or air transport vehicles. In addition, further design constraints and other types of FRP composite materials can be applied during structural optimization.

## Figures and Tables

**Table 1:** Helicopter floor sandwich plate’s technical data [48].

Length	Width	Deflection	Acceleration	Maximum Load	
l	b	$\delta_{max}$	*g*	P	p
(mm)	(mm)	(mm)	(m/sec^2^)	(N)	(Pa)
1500	825	25	9.81·4.5	53510	66217.5

**Table 2:** Boundary conditions for a simply supported sandwich plate of a helicopter floor.

Coefficient for Bending Deflection	Coefficient for Shear Deflection	Moment for Maximum Bending	Force for Maximum Shear	Factor for Buckling
$K_{b}$	$K_{s}$	M	F	β
$\frac{5}{384}$	$\frac{1}{8}$	$\frac{Pl}{8}$	$\frac{P}{2}$	1

**Table 3:** Engineering properties of facing material construction [49].

Type of Layers	Tension/Compression Strength (MPa)	Tension/Compression Modulus of Elasticity (GPa)	Poisson’s Ratio (–)	Cured Ply Thickness (mm)	Weight/Ply (kg/m^2^)
Woven Glass Fiber Phenolic Resin	400/360	20/17	0.13	0.25	0.47
Woven Glass Fiber Epoxy Resin	600/550	20/17	0.13	0.25	0.47
Woven Carbon Fiber Epoxy Resin	800/700	70/60	0.05	0.3	0.45

**Table 4:** Engineering properties for FRP honeycomb core [50].

Characteristics		Compression		Plate Shear			
Density	Cell Dimension	Stabilized		Longitudinal Direction		Transverse Direction	
		Strength	Modulus	Strength	Modulus	Strength	Modulus
(kg/m^3^)	(mm)	(MPa)	(MPa)	(MPa)	(MPa)	(MPa)	(MPa)
104.12	6.35	8.14	828	4	159	2.28	90

**Table 5:** Theoretical results for a sandwich plate of the helicopter floor consisting of composite honeycomb core (fiberglass/phenolic resin) and composite material face sheets with different numbers of layers and fiber orientations using the Excel Solver program (GRG Nonlinear Algorithm).

Type of Face Sheets:	(1) Phenolic Woven Glass Fiber	$W_{min}$	$t_{f,opt}$	$t_{c,opt}$
Layers’ Number and Fiber Orientations:		kg	mm	mm
4 (0°, 90°, 90°, 0°) Optimum value		22.133	1	136
**Type of face sheets:**	**(2) Epoxy woven glass fiber**	$W_{min}$	$t_{f,opt}$	$t_{c,opt}$
**Layers’ number and fiber orientations:**		kg	mm	mm
4 (0°, 90°, 90°, 0°) Optimum value		22.133	1	136
**Type of face sheets:**	**(3) Epoxy woven carbon fiber**	$W_{min}$	$t_{f,opt}$	$t_{c,opt}$
**Layers’ number and fiber orientations:**		kg	mm	mm
**2 (0°, 90°) Optimum value**		**14.486**	**0.6**	**95**
**Type of face sheets:**	**(4) Hybrid composite**	$W_{min}$	$t_{f,opt}$	$t_{c,opt}$
**Layers’ number and fiber orientations:**		kg	mm	mm
4 (0°, 90°, 90°, 0°) Optimum value		15.475	1.1	85

**Table 6:** Theoretical results for a sandwich plate of the helicopter floor consisting of composite honeycomb core (fiberglass/phenolic resin) and composite material face sheets with different numbers of layers and fiber orientations using the Matlab program (Interior Point Algorithm).

Type of Face Sheets:	(1) Phenolic Woven Glass Fiber	$W_{min}$	$t_{f,opt}$	$t_{c,opt}$
Layers’ Number and Fiber Orientations:		kg	mm	mm
4 (0°, 90°, 90°, 0°) Optimum value		22.127	1	136
**Type of face sheets:**	**(2) Epoxy woven glass fiber**	$W_{min}$	$t_{f,opt}$	$t_{c,opt}$
**Layers’ number and fiber orientations:**		kg	mm	mm
4 (0°, 90°, 90°, 0°) Optimum value		22.127	1	136
**Type of face sheets:**	**(3) Epoxy woven carbon fiber**	$W_{min}$	$t_{f,opt}$	$t_{c,opt}$
**Layers’ number and fiber orientations:**		kg	mm	mm
**2 (0°, 90°) Optimum value**		**14.473**	**0.6**	**95**
**Type of face sheets:**	**(4) Hybrid composite**	$W_{min}$	$t_{f,opt}$	$t_{c,opt}$
**Layers’ number and fiber orientations:**		kg	mm	mm
4 (0°, 90°, 90°, 0°) Optimum value		15.475	1.1	85

**Table 7:** Safety factors for design constraints of helicopter floor sandwich plates.

Constraints	Factor of Safety (FoS) Relating to the 4 Different Face Sheets			
	Phenolic Woven Glass Fiber (0°, 90°, 90°, 0°)	Epoxy Woven Glass Fiber (0°, 90°, 90°, 0°)	Epoxy Woven Carbon Fiber (0°, 90°)	Hybrid Composite (0°, 90°, 90°, 0°)
$D_{11,x}$	1.018	1.018	1.027	1.03
δ	1	1	1	1
$\sigma_{f}$	4.173	6.258	3.71	4.05
$\tau_{c}$	9.608	9.608	6.731	3.984
$\sigma_{f}$	10.302	15.741	12.71	15.582
$P_{b,cr}$	Not Active Constraint			
$P_{cr}$	1.812	1.208	1.3	1.585
$P_{wr,cr}$	2.808	1.671	1.652	3.995
$\sigma_{f,cr}$	1.812	1.208	1.3	1.585

**Table 8:** Annual fuel and carbon savings of the sandwich plate for 1 kg.

1. Fuel Saving	Price	Unit
Weight of fuel desired to transport added 1 kg/h	0.04	kg
Weight of fuel desired to transport added 1 kg/1 year	200	kg
Fuel cost/1000 kg	993	USD
Fuel cost to transport added 1 kg/1 year	199	USD
Weight of lightweight sandwich plate of a helicopter floor	14.473	kg
**2. Carbon Savings**		
Carbon generated/1 kg of fuel	3.1	kg
Carbon generated to transport 1 kg/1 year	620	kg
Cost of carbon per ton	40	USD

**Table 9:** Dimensions of honeycomb sandwich models of helicopter floor.

Dimensions	Length	Span	Width	Thickness of Honeycomb Core	Thickness of Face Sheet	Load
Face Sheets	l	s	b	$t_{c}$	$t_{f}$	P
	(mm)	(mm)	(mm)	(mm)	(mm)	(N)
Phenolic Woven Glass Fiber (0°, 90°, 90°, 0°)	1500	1400	825	136	1	53,510
Epoxy Woven Glass Fiber (0°, 90°, 90°, 0°)				136	1	
Epoxy Woven Carbon Fiber (0°, 90°)				95	0.6	
Hybrid Composite (0°, 90°, 90°, 0°)				85	1.1	

**Table 10:** Numerical results of honeycomb sandwich models of helicopter floor using the Digimat-HC program.

Optimal Forms of Different Face Sheets	δ	$\sigma_{skin}$	$\tau_{c}$
	(mm)	(MPa)	(MPa)
(1) Phenolic Woven Glass Fiber	25.925	104	1.06
(2) Epoxy Woven Glass Fiber	25.925	104	1.06
(3) Epoxy Woven Carbon Fiber	30.335	235	1.14
(4) Hybrid Composite	31.541	198	1.03

## Data Availability

The data presented in this study are available on request from the corresponding author.

## Appendix A. List of Symbols

**Table:**

b	Width	mm
d	Distance between facing skin centers	mm
$D_{11,x}$	Bending stiffness in the global coordinate	N·m
$D_{min}$	Minimum stiffness of a sandwich structure	N·m
$E_{c}$	Young’s modulus of elasticity of the core	GPa
$E_{f}$	Average modulus of elasticity	GPa
$E_{f,x}$	Young’s modulus of elasticity of composite face sheet in x direction	GPa
$E_{f,y}$	Young’s modulus of elasticity of composite face sheet in y direction	GPa
F	Maximum shear force	N
*g*	Acceleration	m/sec^2^
$G_{c}$	Core shear modulus	GPa
$G_{L}$	Core shear modulus in L direction (longitudinal direction)	GPa
$G_{W}$	Core shear modulus in W direction (transverse direction)	GPa
$K_{b}$	Bending deflection coefficient	-
$K_{s}$	Shear deflection coefficient	-
l	Length	mm
M	Maximum bending moment	N·m
$N_{cr}$	Number of epoxy woven carbon fiber laminates	piece
$N_{g}$	Number of epoxy woven glass fiber laminates	piece
$N_{l}$	Number of layers in the laminate	piece
$N_{l,opt}$	The optimum number of layers in the laminate	piece
p	Load per unit area	MPa
P	Applied load	N
$P_{b,cr}$	Overall critical buckling load	N
$P_{cr}$	Critical shear crimping load	N
$P_{wr,cr}$	Skin wrinkling critical load	N
s	Span	mm
${\tilde{S}}_{11}$	Shear stiffness of a composite sandwich structure	N/m
$t_{c}$	Core thickness	mm
$t_{c,opt}$	Optimum core thickness	mm
$t_{cr}$	Lamina thickness of epoxy woven carbon fiber face sheet	mm
$t_{fl}$	Thickness of one layer	mm
$t_{f}$	Face sheet thickness	mm
$t_{f,opt}$	Optimum face sheet thickness	mm
$t_{g}$	Lamina thickness of epoxy woven glass fiber face sheet	mm
$t_{l}$	Lamina thickness	mm
$W_{c}$	Core weight	kg
$W_{f}$	Face sheet weight	kg
$W_{f,cr}$	Weight of epoxy woven carbon fiber face sheets	kg
$W_{f,g}$	Weight of epoxy woven glass fiber face sheets	kg
$W_{min}$	Minimum weight	kg
$W_{t}$	Total weight	kg
β	Buckling factor	-
δ	Deflection	mm
$\delta_{max}$	Maximum deflection	mm
$\theta^{{}^{\circ}}$	Fiber orientation angle	degree
$\rho_{c}$	Core density	kg/m^3^
$\rho_{cr}$	The density of epoxy woven carbon fiber	kg/m^3^
$\rho_{f}$	Face sheet density	kg/m^3^
$\rho_{g}$	The density of epoxy woven glass fiber	kg/m^3^
$\sigma_{f}$	Skin stress	MPa
$\sigma_{fib,cr}$	Intracell buckling critical stress	MPa
$\sigma_{f,x}$	Typical yield strength of the composite face sheet in the x direction	MPa
$\sigma_{f,y}$	Typical yield strength of the composite face sheet in the y direction	MPa
$\sigma_{Num}$	Numerical stress	MPa
$\sigma_{skin}$	Equivalent skin stress	MPa
$\sigma_{wr,cr}$	Skin wrinkling critical stress	MPa
$\tau_{c}$	Core shear stress	MPa
$\tau_{c,y}$	Typical shear stress of the core material in the transverse direction	MPa
$\nu_{c}$	Core Poisson’s ratio	-
$\nu_{12}^{f},\nu_{21}^{f}$	Face sheet Poisson’s ratio	-

## Appendix B

Figure A1, Figure A2, Figure A3 and Figure A4 show the graphical evaluation of the simulation four-point bending test relating to the honeycomb sandwich structures of the helicopter floor using the Digimat-HC software. The results of the simulation are the evaluation of the following parameters: (1) δ: vertical displacement of the structure at the mid-section; (2) $\sigma_{skin}$: equivalent skin stress; (3) $\tau_{c}$: equivalent core shear stress, for the optimum sandwich plates of the helicopter floor.
